# Sports and Active Living Are Medicine, and Education, Happiness, Performance, Business, Innovation, and Culture…for a Sustainable World

**DOI:** 10.3389/fspor.2019.00001

**Published:** 2019-04-24

**Authors:** Gregoire P. Millet, Richard Giulianotti

**Affiliations:** ^1^Institute of Sport Sciences, University of Lausanne, Lausanne, Switzerland; ^2^School of Sport, Exercise and Health Sciences, Loughborough University, Loughborough, United Kingdom; ^3^Department of Sports, Physical Education and Outdoor Studies, University of Southeast Norway, Bo, Norway

**Keywords:** injury prevention, physical education, elite sport, sport sociology, sport marketing, sport medicine, sport technology

## Sports and Active Living are Medicine

About 30% of adults worldwide (Hallal et al., 2012) do not meet the minimum World Health Organization recommendation on daily activity (World Health Organization, 2010). The inactivity starts in adolescence with a high proportion (80%) of inactive adolescents [aged 13–15 years and doing less than the WHO-recommended minimum of 60 min daily of physical activity (PA)] (Hallal et al., 2012). Physical inactivity leads to many pathologies, from cardio-metabolic disorders to cardiovascular diseases (Booth et al., 2017). Physical inactivity is now the fourth leading cause of death worldwide (Kohl et al., 2012). The deleterious effects of physical inactivity on health are now well-established for inducing many pathologies, from cardio-metabolic disorders to cardiovascular diseases (van der Ploeg et al., 2012; Booth et al., 2017).

To counterbalance these effects, a pill exists with many pharmacological effects: Physical Activity; i.e., Sports and Active Living (Imbeault, 2007; The exercise pill, 2008; Church and Blair, 2009; Lobelo et al., 2014). Higher PA levels are associated with a lower risk of death and cardiovascular events, irrespective of country income (Arem et al., 2015; Lear et al., 2017). Even a very moderate dose brings benefits for health: low PA loads, even below the WHO minimum recommendations, can provide major benefits; notably, running 5–10 min per day at slow speeds was associated with a considerably reduced risk of cardiovascular death (Lee et al., 2014). However, there are some additional benefits of a higher dose of PA as shown by an inverse relationship between total PA and mortality (Paffenbarger et al., 1993; Lee et al., 1995). However, badly prescribed exercise (too often, too intense, too long, not individualized or adapted) can hurt and may be worse than inactivity (Eijsvogels and Thompson, 2015), leading to musculo-skeletal injuries, pathologies (such as rhabdomyolysis) (Eichner, 2017), or addiction (Hausenblas et al., 2017). We therefore need to know more about injury prevention (Silva et al., 2018) and the different mechanisms of injury occurrence such training load (Soligard et al., 2016; Gabbett and Whiteley, 2017) or biomechanics (Hewett and Bates, 2017).

A key message is that it is never too late to start an active life. Regular exercise has multi-system antiaging effects. Regular endurance exercise promotes longevity through pathways common to effects of caloric restriction (Lanza et al., 2008): regular endurance exercise partly normalizes age-related mitochondrial dysfunction. In elderly patients, PA has a positive effect on cognitive flexibility, depressive symptoms, and sleep disorders, which can be signs of an ongoing neurodegenerative process as Alzheimer's disease and Parkinson's disease (Lerche et al., 2018). This again highlights the role of PA as a disease modifier.

## Sports and Active Living are Education

Sport and physical activity have long-standing ties with education. In Ancient Greece and Rome, for example, physical education for males was directed toward cultivating the perfect human body and training for military purposes (Fuld, 1907). In modern times, the development and spread of many sports and physical disciplines—as diverse as football and rugby codes, gymnastics, cricket, boxing, track and field, judo—are rooted in their practice in schools, colleges and universities in different parts of the world (Mangan, 1986). The subsequent embedding of physical education in modern school curricula reflects state, scientific and practitioner focus on the educational and health benefits of physical activity (Bailey et al., 2009).

Scientific research indicates that physical activity (PA) and cardiorespiratory fitness are associated with a variety of health benefits for children and adolescents (Janssen and LeBlanc, 2010). In addition to the benefits on physical and mental health, studies in neuroscience have shown that PA has positive effects on brain structure and function (Chaddock et al., 2012). Exercise interventions thus have a positive effect on the cognitive performance of children (Hill et al., 2011). The level of PA is positively associated with cognition and academic achievement in children (Lees and Hopkins, 2013) and adolescents (Esteban-Cornejo et al., 2015). Children with higher cardiorespiratory fitness are more successful academically (Marques et al., 2018).

However, during the transition from childhood to adolescence, a marked decrease occurs in the proportion of individuals who meet minimum PA recommendations. Physical education (PE) experiences may contribute to this change. Among many factors, one of the most potentially influential is the exposure to, and the experiences associated with PE at school (Mura et al., 2015). For many children, PE may be the only opportunity for regular PA, making it essential that it is delivered in a manner that encourages lifelong PA. Sadly, during adolescence, for many children, their attitudes toward PE begin to become more negative (Mercier et al., 2017). Childhood memories of PE are associated with PA attitude, intention, and sedentary or active behavior in adulthood (Ladwig et al., 2018).

Social scientists have long highlighted how social divisions—notably class, gender, ethnicity, and (dis)ability—shape young people's access to, and experiences of, sport and PA in educational settings (Evans, 1993). Thus, in market-based societies, private schools, and elite universities offer far more extensive and better quality sport training and facilities than state schools can provide; more broadly, in most societies, girls, ethnic minority groups, and people with disabilities, continue to experience different types of marginalization and lack of sporting provisions within educational settings (Stidder and Hayes, 2013; Collins and Kay, 2014).

At the same time, schools, NGOs, governmental bodies, and sport organizations have turned to use sport and physical activities as a hook for drawing young people into education and training, and as tools for educational messages in different areas of personal and social development, such as on health, gender empowerment, and promoting the inclusion of ethnic minorities (Holt, 2016; Collison et al., 2019). Some research has argued for “critical pedagogies” within physical education, to promote critical reflection and social change among young learners (Fitzpatrick, 2013).

## Sports and Active Living are Happiness

There is a large body of evidence that regular exercise improves emotional experiences and well-being (Nagamatsu et al., 2014; Hallgren et al., 2016). Immediately after engaging in PA, participants report feeling more positive (e.g., more energy and vigor) (Reed and Ones, 2006) and less negative (e.g., less fatigue and anxiety) (Youngstedt, 2010). Exercise is also known as an effective counterbalance measure against depression. PA is commonly recommended as part of treatment for those suffering from emotional disorders (Walsh, 2011). In elderly patients, a larger remission of latelife major depression was observed when following a pharmacological treatment combined with aerobic exercise, showing that physical exercise is safe and adds effective antidepressant effects (Belvederi Murri et al., 2015).

Moreover, feeling good after PA may promote more PA over time and subsequently positive Emotions that are important predictors of life “resources”—psychological (e.g., environmental mastery), social (e.g., social bonds), or cognitive (e.g., creativity)—(Fredrickson, 1998). In other words, being active helps to keep an active life in many areas.

## Sports and Active Living are Performance

Investigating the mechanisms and applications of performance enhancement in athletes is an important area in sport sciences. There is a large cohort of specialists (coach, exercise physiologist, psychologist, data analyst, physiotherapist, conditioning coach, mental trainer, sport physician, biomechanist, nutritionist, recovery specialist, …) working as the “team behind the team” to enhance sport performance, prevent decrements in performance and enhance recovery of athletes. These multi-disciplinary staff members try to follow an evidence-based approach integrating the best relevant research evidence into the decision-making process for the service delivery to athletes.

Unfortunately, there are remaining gaps between scientists and practitioners with difficulties for implementing scientific results into practice (Stoszkowski and Collins, 2016; Halperin et al., 2018). One effective way to bridge the gap between exercise scientists and coaches may be to implement qualitative research (Halperin, 2018). A good example of fruitful collaboration might arise from case studies, especially for reporting exceptional performance or athlete characteristics (Schmitt et al., 2016).

## Sports and Active Living are Business and Innovation

Sport, tourism and other fields of active living are commercial sectors that have grown rapidly over the past few decades. Industry analysts have valued the global sport market at around US$600-700 billion, or around 1% of global GDP (KPMG, 2016); the burgeoning fitness and mind-body industries at US$595 billion (Global Wellness Institute, 2018); and, the global travel and tourism industries at US$8,272 billion (World Travel and Tourism Council, 2018). Sport's commercial value is most clearly demonstrated at the elite level, for example as the world's 50 most valuable clubs—such as Dallas Cowboys (American football), New York Yankees (baseball), and Manchester United and Real Madrid (association football or soccer)—are worth a combined US$137 billion.

The commercial expansion of the sport and active living sectors is manifested in a variety of ways. As global sectors, they increasingly interconnect diverse communities, cities, nations and regions. They encompass a panoply of products and services, not only in sporting events and infrastructure, but also in science and technology, fashion and merchandise, media, education, and even the “happiness” industries (Davies, 2015). They include many fields of employment, such as in sport and leisure management, marketing, business, communication, education, innovation, analytics, and engineering. They produce new technologies such as wearable devices for physical and psychological health, smartphone apps, software for performance analysis, and gaming media. These rapid developments inspire critical investigation and inquiry by academics. For example, wearable technologies raise various issues and concerns regarding constant self-monitoring, obsessions with health, and the confidentiality and scientific quality of data that is generated (Van den Bulck, 2015; Halson et al., 2016; Sperlich et al., 2017; Baron et al., 2018). In addition, their transnational expansion leads to concerns over how sport and active living industries may feed on or exacerbate global inequalities or human rights abuses, for example in the exploitation of employees in sport merchandise factories or on stadium construction sites; and, the decline of local sport clubs and competitions in the global South due to the transnational appeal of glamorous sport events in the global North (Giulianotti and Robertson, 2009; Sage, 2010; Brannagan and Giulianotti, 2015).

## Sports and Active Living are History, Society, Culture, and Politics

Sport and physical culture have the deepest historical roots. Different sporting forms were practiced by ancient civilizations, notably by the Greeks and Romans, as well as the Egyptian, Mesoamerican and other peoples, often as part of their religious rituals (Guttmann, 2004). From the Eighteenth century onwards, the emergence and transnational spread of association football, rugby, cricket, baseball, basketball, and other modern organized sports depended heavily on British and then American international dominance (Holt, 1989; Guttmann, 1994). Modern sports emerged and developed alongside wider processes of modernization, notably the making of modern capitalism, colonialism and post-colonialism, nationalism, conflict and war, international society, industrialization and urbanization, rationalization and bureaucratization, and diverse social divisions along the lines of class, caste, gender, sexuality, “race” and ethnicity, and [dis]ability (Hargreaves, 1994; Carrington, 2010; Gruneau, 2018).

At everyday level, sport and activity groups facilitate the making and remaking of personal and collective identities, and social relations, ties and obligations, across members and participants. At national and transnational levels, sport events often generate high spectatorships and the largest television audiences (Whannel, 2009). Competitive sports also dramatize and, at times, amplify rivalries and oppositions between different competitors, communities and societies (Armstrong and Giulianotti, 2001).

Sports and physical cultures tend to carry strong cultural meanings and aesthetic qualities for their practitioners and audiences, such as in symbolizing identities and communities, or in capturing the “grace” of the body at play (Besnier et al., 2017). Sports and active living enable different societies to display their cultural histories, symbols, values and identities before different audiences, such as through hosting the opening ceremonies at the Olympics and other sport mega-events (Traganou, 2010). They are also a field of growing importance for artists, writers, musicians, dancers, and others working creatively in the humanities and arts.

Sport and active living harbor strong political dimensions, where competing interests come into play, for example, between powerful groups (typically male, white, heterosexual, middle or upper class, global North), nations and corporations which dominate sports, and diverse dominated groups that pursue full sporting participation (Sage, 2010; Giulianotti, 2015). The politics of sport governance have also intensified due to a widening of the array of political stakeholders (such as club owners, athletes, states, corporate sponsors, and fans), and also due to recent corruption scandals in FIFA and other sport bodies (Donnelly, 2015; Jennings, 2015).

Thus, we see that sport and active living interconnect very substantially with these fields, in medicine, education, happiness, performance, business and innovation, history, society, culture, and politics. This journal is committed to identifying and addressing the critical scientific challenges that arise in sport and active living across these diverse fields. Our aim is that, in doing so, the journal will become a prominent vehicle, for advancing global debates across the natural and social sciences, and for developing appropriate recommendations in policy and practice, on sport and active living, in making a more sustainable world.

## Author's Note

It gives us great pleasure to introduce the new journal, *Frontiers in Sports and Active Living*. This open-access journal publishes rigorously peer-reviewed research that advances our understanding of all aspects of sports, physical activity, exercise training; in other words, of active living, of non-sedentary behavior. As Field Chief Editors, we have the enormous benefit of being supported by an outstanding Editorial Board of international researchers.

We are committed to ensuring that this multidisciplinary journal will be at the forefront of disseminating and communicating scientific knowledge and impactful discoveries to researchers, academics, policy-makers, and the public worldwide. To this end, *Frontiers in Sports and Active Living* has developed a number of specialties that clearly outline our research areas. We particularly welcome new ideas and approaches that are likely to shift paradigms in our understandings of sport, exercise, and active living.

The journal will register and explore how sports, exercise, and active living permeate all aspects of society. Being active and mobile is for physical health (Exercise is medicine), for mental health (Exercise is happiness), for learning (Exercise is education), for the economy (Exercise is business), for technological development (Exercise is innovation), and for the environment (Exercise for a clean planet). In exploring these and other themes, which we elaborate on below, the journal seeks to contribute to developing a sustainable world, and in the process will join forces with other Fields within the Frontiers domain.

## Author Contributions

All authors listed have made a substantial, direct and intellectual contribution to the work, and approved it for publication.

### Conflict of Interest Statement

The authors declare that the research was conducted in the absence of any commercial or financial relationships that could be construed as a potential conflict of interest.

## References

[B1] Arem H. Moore S. C. Patel A. Hartge P. Berrington de Gonzalez A. Visvanathan K.. (2015). Leisure time physical activity and mortality: a detailed pooled analysis of the dose-response relationship. JAMA Intern. Med. 175, 959–967. 10.1001/jamainternmed.2015.0533 25844730 PMC4451435

[B2] Armstrong G. Giulianotti R (eds.) (2001). Fear and Loathing in World Football. Oxford: Berg.

[B3] Bailey R. Armour K. Kirk D. Jess M. Pickup I. Sandford R. (2009). The educational benefits claimed for physical education and school sport: an academic review. Res. Pap. Educ. 24, 1–27. 10.1080/02671520701809817

[B4] Baron K. G. Duffecy J. Berendsen M. A. Cheung Mason I. Lattie E. G. Manalo N. C. (2018). Feeling validated yet? A scoping review of the use of consumer-targeted wearable and mobile technology to measure and improve sleep. Sleep Med. Rev. 40, 151–159. 10.1016/j.smrv.2017.12.002 29395985 PMC6008167

[B5] Belvederi Murri M. Amore M. Menchetti M. Toni G. Neviani F. Cerri M.. (2015). Physical exercise for late-life major depression. Br. J. Psychiatry 207, 235–242. 10.1192/bjp.bp.114.150516 26206864

[B6] Besnier N. Brownell S. Carter T. (2017). The Anthropology of Sport. Los Angeles, CA: University of California Press.

[B7] Booth F. W. Roberts C. K. Thyfault J. P. Ruegsegger G. N. Toedebusch R. G. (2017). Role of inactivity in chronic diseases: evolutionary insight and pathophysiological mechanisms. Physiol. Rev. 97, 1351–1402. 10.1152/physrev.00019.2016 28814614 PMC6347102

[B8] Brannagan P.M. Giulianotti R. (2015). Soft power and soft disempowerment: Qatar, global sport and football's 2022 World Cup finals. Leisure Stud. 34, 703–719. 10.1080/02614367.2014.964291

[B9] Carrington B. (2010). Race, Sport and Politics. London: Sage.

[B10] Chaddock L. Erickson K. I. Prakash R. S. Voss M. W. VanPatter M. Pontifex M. B.. (2012). A functional MRI investigation of the association between childhood aerobic fitness and neurocognitive control. Biol. Psychol. 89, 260–268. 10.1016/j.biopsycho.2011.10.017 22061423

[B11] Church T. S. Blair S. N. (2009). When will we treat physical activity as a legitimate medical therapy…even though it does not come in a pill? Br. J. Sports Med. 43, 80–81. 10.1136/bjsm.2008.053850 18927165

[B12] Collins M. Kay T. (2014). Sport and Social Exclusion. London: Routledge.

[B13] Collison H. Darnell S. Giulianotti R. Howe P.D. (eds.) (2019). The Routledge Handbook of Sport for Development and Peace. London: Routledge.

[B14] Davies W. (2015). The Happiness Industry: How the Government and Big Business Sold Us Well-being. London: Verso.

[B15] Donnelly P. (2015). What if the players controlled the game? Dealing with the consequences of the crisis of governance in sports. Eur. J. Sport Soc. 12, 11–30. 10.1080/16138171.2015.11687954

[B16] Eichner E. R. (2017). Exercise is medicine, but the dosing can be dicey. Curr. Sports Med. Rep. 16, 59–60. 10.1249/JSR.0000000000000339 28282346

[B17] Eijsvogels T. M. Thompson P. D. (2015). Exercise is medicine: at any dose? JAMA 314, 1915–1916. 10.1001/jama.2015.10858 26547459

[B18] Esteban-Cornejo I. Tejero-Gonzalez C. M. Sallis J. F. Veiga O. L. (2015). Physical activity and cognition in adolescents: a systematic review. J. Sci. Med. Sport 18, 534–539. 10.1016/j.jsams.2014.07.007 25108657

[B19] Evans J. (ed.). (1993). Equality, Education, and Physical Education. London: Routledge.

[B20] Fitzpatrick K. (2013). Critical Pedagogy, Physical Education and Urban Schooling. New York, NY: Peter Lang.

[B21] Fredrickson B. L. (1998). What good are positive emotions? Rev. Gen. Psychol. 2, 300–319. 10.1037/1089-2680.2.3.300 21850154 PMC3156001

[B22] Fuld L.F. (1907). Physical education in Greece and Rome. Am. Phys. Educ. Rev. 12, 1–14.

[B23] Gabbett T. J. Whiteley R. (2017). Two training-load paradoxes: can we work harder and smarter, can physical preparation and medical be teammates? Int. J. Sports Physiol. Perform. 12, S250–S254. 10.1123/ijspp.2016-0321 27736264

[B24] Giulianotti R. (2015). Sport: A Critical Sociology. Cambridge: Polity.

[B25] Giulianotti R. Robertson R. (2009). Globalization and Football. London: Sage. 10.1111/j.1468-4446.2004.00037.x 15663424

[B26] Global Wellness Institute (2018). The Global Wellness Economy Monitor. Available online at: https://globalwellnessinstitute.org/press-room/statistics-and-facts/

[B27] Gruneau R. (2018). Sport and Modernity. Cambridge: Polity.

[B28] Guttmann A. (1994). Games and Empires. New York, NY: Columbia University Press.

[B29] Guttmann A. (2004). Sports: The First Five Millennia, Amherst, MA: University of Massachusetts Press.

[B30] Hallal P. C. Andersen L. B. Bull F. C. Guthold R. Haskell W. Ekelund U.. (2012). Global physical activity levels: surveillance progress, pitfalls, and prospects. Lancet 380, 247–257. 10.1016/S0140-6736(12)60646-1 22818937

[B31] Hallgren M. Vancampfort D. Stubbs B. (2016). Exercise is medicine for depression: even when the “pill” is small. Neuropsychiatr. Dis. Treat. 12, 2715–2721. 10.2147/NDT.S121782 27822043 PMC5087774

[B32] Halperin I. (2018). Case studies in exercise and sport sciences: a powerful tool to bridge the science-practice gap. Int. J. Sports Physiol. Perform. 13, 824–825. 10.1123/ijspp.2018-0185 29929417

[B33] Halperin I. Vigotsky A. D. Foster C. Pyne D. B. (2018). Strengthening the practice of exercise and sport-science research. Int. J. Sports Physiol. Perform. 13, 127–134. 10.1123/ijspp.2017-0322 28787228

[B34] Halson S. L. Peake J. M. Sullivan J. P. (2016). Wearable technology for athletes: information overload and pseudoscience? Int. J. Sports Physiol. Perform. 11, 705–706. 10.1123/IJSPP.2016-0486 27701967

[B35] Hargreaves J. (1994). Sporting Females. London: Routledge.

[B36] Hausenblas H. A. Schreiber K. Smoliga J. M. (2017). Addiction to exercise. BMJ 357:j1745. 10.1136/bmj.j1745 28446435

[B37] Hewett T. E. Bates N. A. (2017). Preventive biomechanics: a paradigm shift with a translational approach to injury prevention. Am. J. Sports Med. 45, 2654–2664. 10.1177/0363546516686080 28199800 PMC6405413

[B38] Hill L. J. Williams J. H. Aucott L. Thomson J. Mon-Williams M. (2011). How does exercise benefit performance on cognitive tests in primary-school pupils? Dev. Med. Child Neurol. 53, 630–635. 10.1111/j.1469-8749.2011.03954.x 21649650

[B39] Holt N.L. (ed.). (2016). Positive Youth Development Through Sport. London: Routledge.

[B40] Holt R. (1989). Sport and the British. Oxford: Oxford University Press.

[B41] Imbeault P. (2007). The unswallowed pill: physical activity. Appl. Physiol. Nutr. Metab. 32, 305–306. 10.1139/h06-062 17486173

[B42] Janssen I. LeBlanc A. G. (2010). Systematic review of the health benefits of physical activity and fitness in school-aged children and youth. Int. J. Behav. Nutr. Phys. Act. 7:40. 10.1186/1479-5868-7-40 20459784 PMC2885312

[B43] Jennings A. (2015). The Dirty Game. London: Arrow.

[B44] Kohl H. W. III. Craig C. L. Lambert E. V. Inoue S. Alkandari J. R. Leetongin G.. (2012). The pandemic of physical inactivity: global action for public health. Lancet 380, 294–305. 10.1016/S0140-6736(12)60898-8 22818941

[B45] KPMG (2016). The Business of Sport. Delhi: KPMG.

[B46] Ladwig M. A. Spyridoula V. Panteleimon E. (2018). “My best memory is when i was done with it”: PE memories are associated with adult sedentary behavior. Transl. J. Am. Coll. Sports Med. 3, 119–129.

[B47] Lanza I. R. Short D. K. Short K. R. Raghavakaimal S. Basu R. Joyner M. J.. (2008). Endurance exercise as a countermeasure for aging. Diabetes 57, 2933–2942. 10.2337/db08-0349 18716044 PMC2570389

[B48] Lear S. A. Hu W. Rangarajan S. Gasevic D. Leong D. Iqbal R.. (2017). The effect of physical activity on mortality and cardiovascular disease in 130 000 people from 17 high-income, middle-income, and low-income countries: the PURE study. Lancet 390, 2643–2654. 10.1016/S0140-6736(17)31634-3 28943267

[B49] Lee D. C. Pate R. R. Lavie C. J. Sui X. Church T. S. Blair S. N. (2014). Leisure-time running reduces all-cause and cardiovascular mortality risk. J. Am. Coll. Cardiol. 64, 472–481. 10.1016/j.jacc.2014.04.058 25082581 PMC4131752

[B50] Lee I. M. Hsieh C. C. Paffenbarger R. S. Jr. (1995). Exercise intensity and longevity in men. The Harvard Alumni Health Study. JAMA 273, 1179–1184. 10.1001/jama.1995.03520390039030 7707624

[B51] Lees C. Hopkins J. (2013). Effect of aerobic exercise on cognition, academic achievement, and psychosocial function in children: a systematic review of randomized control trials. Prev. Chronic Dis. 10:E174. 10.5888/pcd10.130010 24157077 PMC3809922

[B52] Lerche S. Gutfreund A. Brockmann K. Hobert M. A. Wurster I. Sunkel U.. (2018). Effect of physical activity on cognitive flexibility, depression and RBD in healthy elderly. Clin. Neurol. Neurosurg. 165, 88–93. 10.1016/j.clineuro.2018.01.008 29331872

[B53] Lobelo F. Stoutenberg M. Hutber A. (2014). The exercise is medicine global health initiative: a 2014 update. Br. J. Sports Med. 48, 1627–1633. 10.1136/bjsports-2013-093080 24759911

[B54] Mangan J.A. (1986). The Games Ethic and Imperialism: Aspects of the Diffusion of an Ideal. Harmondsworth: Penguin.

[B55] Marques A. Santos D. A. Hillman C. H. Sardinha L. B. (2018). How does academic achievement relate to cardiorespiratory fitness, self-reported physical activity and objectively reported physical activity: a systematic review in children and adolescents aged 6-18 years. Br. J. Sports Med. 52:1039. 10.1136/bjsports-2016-097361 29032365

[B56] Mercier K. Donovan C. Gibbone A. Rozga K. (2017). Three-year study of students' attitudes toward physical education: grades 4-8. Res. Q. Exerc. Sport 88, 307–315. 10.1080/02701367.2017.1339862 28661718

[B57] Mura G. Rocha N. B. Helmich I. Budde H. Machado S. Wegner M.. (2015). Physical activity interventions in schools for improving lifestyle in European countries. Clin. Pract. Epidemiol. Ment Health 11, 77–101. 10.2174/1745017901511010077 25834629 PMC4378026

[B58] Nagamatsu L. S. Flicker L. Kramer A. F. Voss M. W. Erickson K. I. Hsu C. L.. (2014). Exercise is medicine, for the body and the brain. Br. J. Sports Med. 48, 943–944. 10.1136/bjsports-2013-093224 24659507 PMC4330095

[B59] Paffenbarger R. S. Jr. Hyde R. T. Wing A. L. Lee I. M. Jung D. L. Kampert J. B. (1993). The association of changes in physical-activity level and other lifestyle characteristics with mortality among men. N. Engl. J. Med. 328, 538–545. 10.1056/NEJM199302253280804 8426621

[B60] Reed J. Ones D. S. (2006). The effect of acute aerobic exercise on positive activated affect: a meta-analysis. Psychol. Sport Exerc. 7, 477–514. 10.1016/j.psychsport.2005.11.003

[B61] Sage G.H. (2010). Globalizing Sport. London: Taylor and Francis.

[B62] Schmitt L. Regnard J. Auguin D. Millet G. P (2016). Monitoring training and fatigue status with heart rate variability: case study in a swimming Olympic champion. J Fitness Research. 5, 38–45.

[B63] Silva P. V. Kamper S. J. da Cunha Menezes Costa L. (2018). Exercise-based intervention for prevention of sports injuries (PEDro synthesis). Br. J. Sports Med. 52, 408–409. 10.1136/bjsports-2017-098474 29146751

[B64] Soligard T. Schwellnus M. Alonso J. M. Bahr R. Clarsen B. Dijkstra H. P.. (2016). How much is too much? (Part 1) International Olympic Committee consensus statement on load in sport and risk of injury. Br. J. Sports Med. 50, 1030–1041. 10.1136/bjsports-2016-096581 27535989

[B65] Sperlich B. Duking P. Holmberg H. C. (2017). A SWOT analysis of the use and potential misuse of implantable monitoring devices by athletes. Front. Physiol. 8:629. 10.3389/fphys.2017.00629 28928670 PMC5591786

[B66] Stidder G. Hayes S. (eds.) (2013). Equity and Inclusion in Physical Education and Sport. London: Routledge.

[B67] Stoszkowski J. Collins D. (2016). Sources, topics and use of knowledge by coaches. J. Sports Sci. 34, 794–802. 10.1080/02640414.2015.1072279 26222481

[B68] The exercise pill (2008). The exercise pill. Clin. Med. 8, 469–470. 10.7861/clinmedicine.8-4-469 PMC4952958 18724625

[B69] Traganou J. (2010). National narratives in the opening and closing ceremonies of the Athens 2004 Olympic Games. J Sport Soc. Issues 34, 236–251. 10.1177/0193723509360217

[B70] Van den Bulck J. (2015). Sleep apps and the quantified self: blessing or curse? J. Sleep Res. 24, 121–123. 10.1111/jsr.12270 25558955

[B71] van der Ploeg H. P. Chey T. Korda R. J. Banks E. Bauman A. (2012). Sitting time and all-cause mortality risk in 222 497 australian adults. Arch. Intern. Med. 172, 494–500. 10.1001/archinternmed.2011.2174 22450936

[B72] Walsh R. (2011). Lifestyle and mental health. Am. Psychol. 66, 579–592. 10.1037/a0021769 21244124

[B73] Whannel G. (2009). Television and the transformation of sport. Ann. Am. Acad. Polit. Soc. Sci. 625, 205–218. 10.1177/0002716209339144

[B74] World Health Organization (2010). Global Recommendations on Physical Activity for Health. 26180873

[B75] World Travel Tourism Council (2018). World Economic Impact: Travel and Tourism. Available online at: https://www.wttc.org/-/media/files/reports/economic-impact-research/regions-2018/world2018.pdf

[B76] Youngstedt S. D. (2010). Comparison of anxiolytic effects of acute exercise in older versus younger adults. J. Appl. Gerontol. 29, 251–260. 10.1177/0733464809337411 25374437 PMC4217700

